# On becoming and being an international medical educator

**DOI:** 10.5116/ijme.5159.c66c

**Published:** 2013-04-02

**Authors:** Michelle McLean

**Affiliations:** Faculty of Health Sciences and Medicine, Bond University, Gold Coast, Australia

It is almost 15 months since I arrived on Australian soil as the Academic Lead for Problem-based Learning at Bond University, after a five and half year stint in the Middle East. How did a South African end up in Australia via the Middle East, you might well ask? The story of this journey to becoming an “international” medical educator began in April 2005, when my cosy (but not mundane) routine at the University of KwaZulu-Natal (South Africa) was interrupted by an email inviting me to consider applying for a medical education post at the United Arab Emirates (UAE) University. Why would I want to work in the UAE or anywhere else, for that matter? Although being what I considered “well-travelled”, the thought of disrupting my relatively peaceful and comfortable existence in the city in which I was born, schooled and now worked, held little appeal.But, the seed had been sown.... After weighing up the pros and the cons, I took a huge leap of faith and applied for the position, not knowing what lay beyond the next four years or even if I would see out the four years in a new and strange country. My decision was certainly not taken lightly. In fact, it was a gestation of several months, particularly since I knew that if I resigned, considering the South African political landscape, it was unlikely that I would find a similar position on my return. So, leaving family, friends, assets and a lifestyle to which I was accustomed, I landed in Dubai in the height of summer (in excess of 45°C) and was transported to the garden city of Al Ain, in the middle of the Arabian desert. From a professional perspective, it was one of the best decisions I have made. The UAEU faculty comprised mostly expatriate academics and support staff with a wealth of knowledge and experience who too had had to make the often difficult decision to leave “home”, sometimes without their families, in search of, amongst other reasons, a different (or for some, a similar) experience, adventure, research opportunities, a tax-free income or perhaps exiled from their home country because of political strife. At the time of my arrival (June 2006), faculty originated from more than 25 countries. While many were from the region (e.g. Syria, Jordan, Palestine, Iran, Iraq) or from North Africa (e.g. Egypt, Libya, Sudan), others hailed from more distant shores (Canada, Australia, New Zealand, South Africa). Many of us lived in the same “compounds” and so became a close-knit family, sharing our stories of families left behind and caring for our inherited pets (usually cats) who adopted us the minute we moved into our new accommodation. We regularly shared meals and frequently escaped to the desert or the coast to enjoy the splendour of outdoors, particularly in the cooler winter months. Colleagues soon became friends, often cemented by our common origins and the familiar “culture” of our heritage. Although continents now separate us, the bonds that united us when our paths crossed on foreign shores still remain, thanks largely to social media.Contract work away from home often comes with perks. The UAEU’s generous leave and research support, for example, allowed me to travel extensively both professionally and for leisure. I soon came to realise that Dubai and Abu Dhabi are the gateways to a significant part of the globe, with Africa, Europe and Asia readily accessible in a few hours. The UAE position was the springboard for developing a network of collaborators both locally and further afield. Either as a key note speaker at conferences or as an external examiner, I was able to travel to places I would never have dreamed of visiting had I stayed in South Africa: Indonesia, Syria, Saudi Arabia (which was interesting as the only invited female presenter), Bahrain and Sudan. For well-earned vacations, I was able to explore the wonders of the Maldives, Sri Lanka, Bhutan, India, Jordan, Oman, Malta, Cyprus, Tanzania, Kenya and Zanzibar. Sadly, however, due to the “Arab Spring”, some places remain on my bucket list: Yemen, including Socotra and Algeria. Travelling on a South African passport, my little red automobile went into autopilot mode on the numerous Al Ain-Abu Dhabi trips to embassies and consulates. A bumper 30-page passport was needed as my standard 10-page (still valid for several years) was soon full of expensive visas.Working abroad for the first time in a different culture and considering the soul-searching that had culminated in the decision to leave my homeland for an unknown future, sparked my curiosity. What drives individuals to work abroad, to become international medical educators? What are the issues that they face, particularly if the culture is far removed from their own? In collaboration with fellow international medical educators, some of whom I had yet to meet, we embarked on a study to explore what it really means to be an “international” educator. Starting with colleagues in the Middle East and then extending the study to capture an international audience, we advertised our survey through our global network of fellow educators. The inclusion criterion was simple: Are you or have you worked outside your home country as a medical educator? Our analysis of largely qualitative data is “work in progress”. I am, however, able report some of our preliminary findings. Eighty-nine individuals, representing 29 countries (7 continents), ranging in age from 28 to 82 (average: 51 years), 40% being female, responded. Eighty-four percent reported being medically qualified. English was the home language of just over 60% of the respondents, with the majority claiming to speak at least one other language. When asked which country they considered home, 30% identified the UK, with next largest group of respondents (15%) falling into “difficult to say” category, which included comments such as “I don’t feel attached to either country” or providing an answer with multiple countries. Of interest was the use of “now” by a number of respondents to indicate the country in which they were currently working as “home”, transcending the idea of “home” being the country of birth. From my personal experience, currently working in a second country since leaving South Africa, and having just submitted my Australian permanent residency application, “home” is beginning to blur.In 2006, Ron Harden described his vision of the future of medical education – transnational was the term he used. In such a scenario, the educators and students would be international and the curriculum would be “global”.^1^ In essence, there would be no fences. From personal experience both in the UAE and in Australia, with the status of “international”, “culture” needs to be added to the equation. My move to the Arabic and Islamic UAE “culture” was nothing short of a cultural awakening. Despite all my experiences in a diverse South Africa (11 national languages), I felt like a cultural neonate as I came to understand the intertwined reality of religion and culture and how this impacts on the day-to-day activities of the men and women in the society. Little was, however, done by the institution to prepare international faculty for the gender-segregated nature of the Emirate society, leaving me to bumble through this cultural wilderness. Like several of my expatriate colleagues, while embracing our new experiences, we were often unsure of where the line was – what we could and could not do or say. Our UAEU study identified the difficulty some expatriates experienced in adjusting to a different set of professional values, often challenging their Western patient-centred approach to care. In traditional Bedouin culture, for example, women generally do not make their own medical decisions. These usually rest with the eldest male or with the males in the family. Very quickly, I found myself consulting the culture gurus to understand some of these intricacies.^2^^-^^3^And so, after five and half years in the Middle East, halfway through my second four-year contract, I accepted a position at Bond. Back to my comfort zone, I imagined. Australia, like South Africa, is part of the Commonwealth, so how different will the “culture” be? Soon after arriving, my preconceptions were immediately challenged. Bond is a “private” (non-profit) university, with a considerable part of its income derived from international students. The medical faculty is, however, the exception, with applicants needing to be Australian or New Zealand residents or citizens. But, most medical students are not what one imagines as the typical Australian. Much to my surprise, most medical students were either born elsewhere and are first or second generation Australians. Many are from culturally diverse parents (e.g. Chinese and Australian). The mix is eclectic, with many proudly of Asian (e.g. Vietnamese, Korean, Chinese) and South East Asian (e.g. Sri Lankan, Indian) heritage. For good measure, add a few students of Arabic and African. A fair number of the academic staff too is international. Most have or are in the process of becoming Australian residents or citizens (myself included) and represent several countries and continents: UK, Turkey, Germany, Greece, New Zealand, USA, and South Africa. I think the Bond scenario is very close to Harden’s (2006) transnational vision of medical education. The 2012 QS university rankings list Bond University as 50^th^ in terms of its international flavour.^4^ With Bond’s renewed medical curriculum to roll out in May 2013, I have been keeping beady eye on the content and the scope of the patients in the problem-based learning cases, to ensure that sufficient attention is afforded to cultural diversity and global health.^5^Am I “home”? While Australia is the planned resting place (for the unforeseen future), there are still too many ties to South Africa. I don’t believe that it is possible to forget where you spent your formative years. Perhaps because “home” was Africa, an enigmatic continent with a surprise around every corner, it is harder to relinquish roots. In reality, rather than view ourselves as international medical educators, perhaps we should consider ourselves as global citizens - global in that we travel and often work outside of our place of birth and citizens in that what we do as medical educators is for the advancement of humankind, irrespective of the origins of our students and patients.
